# MDM2 inhibitor APG-115 synergizes with ABT-199 to induce cell apoptosis in chronic lymphocytic leukemia

**DOI:** 10.3389/fphar.2024.1441383

**Published:** 2024-07-31

**Authors:** Ying Cui, Xiaoya Shao, Haiping Yang, Jingyi Xin, Yuanyuan Liu, Mingxiao Zhang, Chuanyue Sun, Ge Chen, Guomin Shen, Xueqiong Meng, Yixiang Chen

**Affiliations:** ^1^ Henan International Joint Laboratory of Thrombosis and Hemostasis, School of Basic Medical Science, Henan University of Science and Technology, Luoyang, China; ^2^ The Second Affiliated Hospital, Henan University of Science and Technology, Luoyang, China; ^3^ The First Affiliated Hospital, Henan University of Science and Technology, Luoyang, China; ^4^ Luoyang Key Laboratory of POCT Diagnosis Technology, Luoyang, China; ^5^ Zhongyuan Scholars Workstation of Henan, Luoyang Polytechnic, Luoyang, China; ^6^ Henan Engineering Research Center of Key Immunological Biomaterials, Luoyang Polytechnic, Luoyang, China

**Keywords:** chronic lymphocytic leukemia, APG-115, apoptosis, ABT-199, combination

## Abstract

Although clinical outcomes in chronic lymphocytic leukemia (CLL) have greatly improved with several approved small molecular inhibitors, acquired resistance does occur, leading to disease progression and eventual death. Thus, the effort to explore novel inhibitors and combination therapeutic regimens is needed. The inhibition of MDM2-p53 interaction to restore p53 function has been regarded as a potential strategy for treating different cancers. We investigated the effects of novel MDM2 inhibitor APG-115 in CLL. We found that APG-115 treatment upregulated the expression of p53, MDM2, and p21 at the mRNA and protein level. APG-115 inhibited cell proliferation, induced apoptosis, and arrested the cell cycle at G0/G1 stage. Moreover, APG-115 inhibited the expression of BCL-2, BCL-xL, and MCL-1, and suppressed the activation of AKT and ERK signaling pathways. APG-115 combined with the BCL2 inhibitor, ABT-199 (venetoclax), led to further inhibition of the expression of BCL-2 family anti-apoptotic proteins and consequently enhanced cell death. Collectively, this study demonstrates that APG-115 activates p53 and thus inhibits multiple pro-survival mechanisms, which provides a rational explanation for APG-115 efficiency in inducing cell apoptosis in CLL. The synergistic effect of APG-115 with ABT-199 suggested a potential combination application in CLL therapy.

## 1 Introduction

Chronic lymphocytic leukemia (CLL) is the most common leukemia in the Western world (Albi et al., 2022). In recent years, with the in-depth study of the pathogenesis of CLL and the clinical application of small molecule targeted drugs such as B-cell lymphoma-2 (BCL-2) inhibitors, the therapy of CLL has significantly improved (Woyach et al., 2014; Cervantes-Gomez et al., 2015). Although clinical outcomes are improved with these inhibitors, acquired resistance occurs, eventually leading to an incurable disease progression (Reiff et al., 2018; Kittai and Woyach, 2019; Liu et al., 2022). Thus, the effort to explore novel inhibitors and combination approaches is needed.

p53, a tumor suppressor, is crucial in controlling cell tumorigenesis and cancer development by regulating cell cycle, DNA repair, and anti-apoptotic protein expression (Maclaine and Hupp, 2009). However, in nearly all human malignant tumors, the p53 signaling pathway is often abnormal. The high expression of mouse double minute 2 homolog (MDM2) is a prominent factor impairing p53 function (Fang et al., 2021). MDM2 controls the activity of p53 through E3 ligase and proteasome-mediated degradation and p53 transcriptional activity inhibition (Haupt et al., 1997; Honda et al., 1997; Shangary and Wang, 2008). Oncogenic MDM2, commonly over-expressed in various human cancers, can potentially enhance carcinogenesis and resistance to apoptosis. As an effective inhibitor of p53 activation, targeting the MDM2-p53 interaction by small molecules to reactivate p53 has emerged as an attractive therapeutic strategy for cancer therapy, especially for tumor cells with wild-type or functional p53 (Shangary and Wang, 2008). The TP53 gene mutations are rare in CLL cells, accounting for about 5%–10% of patients at presentation, and most CLL patients retain a functional p53 (Gaidano et al., 1991; Cordone et al., 1998; Samuel et al., 2016). In addition, more than 28% of the CLL patients had more than 10-fold higher levels of MDM2 gene expression than that in normal B-cell (Watanabe et al., 1994). The over-expression of MDM2 plays a role in tumorigenicity and disease progression of CLL (Kojima et al., 2006; Saddler et al., 2008). In recent years, several MDM2 small molecules have been developed and evaluated in different malignancies (Konopleva et al., 2020). MDM2 inhibitors also have shown the ability to induce CLL cell apoptosis, particularly for the cells with wild-type p53 (Kojima et al., 2006; Saddler et al., 2008). Preclinical and clinical studies of MDM2 inhibitors have supported the viability of this approach to mediate anti-tumor activity (Andreeff et al., 2016; Tisato et al., 2017; Konopleva et al., 2020).

APG-115 (Alrizomadlin) is a newly developed, orally active, highly selective small molecule inhibitor of MDM2 with a high binding affinity for MDM2. It can destabilize MDM2-p53 complexes and restore p53 activity (Aguilar et al., 2017). Recently, it has been reported that APG-115 alone or combined with other compounds exerts substantial antitumor activity in several cancer cells (Luo et al., 2020; Fang et al., 2021; Zhai et al., 2023). In addition, APG-115 also synergizes with PD-1 blockade, synergistically enhancing antitumor immunity in the tumor microenvironment (Fang et al., 2019). APG-115 enhanced radiation-induced apoptosis and cell cycle arrest, thus enhancing the antitumor effect of radiotherapy in gastric adenocarcinoma cells (Yi et al., 2018). However, whether APG-115 has an antitumor effect in CLL has not been investigated. ABT-199 (venetoclax), a highly selective and effective BCL-2 inhibitor, has shown remarkable efficacy in treating CLL (Lasica and Anderson, 2021). However, despite its effectiveness, drug resistance, toxicity, burden on the patients, and compromised compliance considerably limit prolonged monotherapy of venetoclax. Thus, drug combinations have become a promising strategy in CLL therapy (Eichhorst et al., 2023; Shadman, 2023). Most recently, APG-115 combined with lisaftoclax showed a synergic effect on venetoclax-resistant Acute Myeloid Leukemia (AML) and Acute Lymphoblastic Leukemia (ALL) (Zhai et al., 2023). This suggested a potential clinical benefit of the combination of APG-115 and BCL-2 inhibitor on CLL patients, particularly those resistant to ABT-199. In the present study, we investigated the efficiency of APG-115 alone and in combination with ABT-199 in CLL and their possible mechanisms. We found that APG-115 upregulated p53 expression and its downstream targeted genes inhibited the expression of BCL-2 family anti-apoptotic proteins and suppressed the activation of AKT and ERK pro-survival signaling pathways. APG-115 induced apoptosis and the combination with ABT-199 exhibited a synergistic pro-apoptotic effect by further inhibiting the expression of BCL-2, BCL-xL, and MCL-1, suggesting a potential application in CLL therapy.

## 2 Materials and methods

### 2.1 Cell culture and reagents

EHEB (TP53-wild type) CLL cell line, a chronic B-cell leukemia cell was cultured in RPMI-1640 medium (Corning) supplemented with 10% (v/v) fetal bovine serum (FBS) (ABW) and 50 µ/mL penicillin, 50 mg/mL streptomycin (Solarbio) at 37°C incubator containing 5% CO_2_. Peripheral blood samples were obtained from CLL (*TP53*-wt) patients attending clinics at First Affiliated Hospital, Henan University of Science and Technology, following informed consent and approval from the local research ethics committee (Supplementary Table S1). Primary cells were isolated by STEMCELL 19,664 B-CLL Sorting Kit EasySepTM Direct Human B-CLL Cell Isolation Kit (Canada), cultured with RPMI-1640 standard medium plus 10 ng/mL CD40L and IL-4 (Peprotech). APG-115 was obtained from Suzhou Ascentage Pharma Co., Ltd. ABT-199 was a product of MCE. Inhibitors were dissolved in dimethyl sulfoxide (DMSO) (Sigma) to make a stock solution for *in vitro* experiments.

### 2.2 Assessment of cell proliferation, apoptosis and cell cycle

Cell proliferation was assessed by the CellTiter 96 A Queous One Solution Cell Proliferation MTS Assay (Promega), following the manufacturer’s instructions as previously described (Chen et al., 2016). The absorbance was read by a microplate reader (Bio-TED) at 490 nm. This experiment was performed in triplicate and repeated at least three times. Cell apoptosis was determined with an Annexin-V-propidium iodide (PI) apoptosis detection kit (Biolegend) by flow cytometry assays. Cells were harvested and stained with 5 µL Annexin V-FITC and 10 µL Propidium Iodide (PI) solution for 15 min at room temperature (25°C) in the dark. Percentages of apoptotic cells were determined by flow cytometry using a FACS Canto II cytometer (BD Biosciences), and the results were analyzed by FlowJo software. Experiments were performed in duplicate and repeated three independent times. The synergy of drug combinations was analyzed by Compusyn software (ComboSyn, Inc.), which allowed us to compute the combination index (CI). For the assessment of the cell cycle, cells were harvested and fixed with cold 70% ethanol and store at 4°C or at −20°C for at least 2 h. After this, cells were washed in PBS buffer and digested by 0.2–0.5 μg/mL RNase (Solarbio) at 37°C for 30 min protected from light. Cells were incubated with 10 μg/mL PI solution at 37°C for 30 min in the dark before being analyzed. The cell cycle profiles were determined using a FACS Canto II cytometer (BD Biosciences) and analyzed with ModFitLT software.

### 2.3 Western blotting

The whole cell proteins were lysed with SDS sample buffer (Solarbio) containing a 1% protease inhibitor cocktail and 1% phosphatase inhibitor cocktail (Sigma). Protein samples were boiled for 5–10 min before separating with SDS-PAGE, electrophoresis was performed at 120 V for 1.5 h, and proteins were then transferred to nitrocellulose (NC) membranes (GE Healthcare) at 0.35 A for 1.5 h in the cold room. NC membranes were blocked with 5% (w/v) BSA with PBS containing 0.05% Tween-20 at room temperature for 1 h. NC membranes were incubated with specific primary antibodies (dilution ratio at 1:1,000) at room temperature for 1 h, or at 4°C overnight, and then incubated with the appropriate HRP-conjugated secondary antibodies (1:5,000 dilution) for 1 h at room temperature with extensive washing in PBS-T between each step. The antibodies of p53, AKT, phospho-AKT (Ser473), ERK, phospho-ERK(Thr202/Tyr204), caspase-3 and PARP were bought from Cell Signaling Technology (CST); BCL-xL was a product of Solarbio; MCL-1 was purchased from Santa Cruz; MDM2 came from Elabscience; p21 and cleaved-caspase-3 were from Proteintech; BAX, and anti-rabbit and anti-mouse secondary antibodies were obtained from Servicebio; BCL-2 and β-actin were purchased from Bioss. The NC membranes were incubated with ECL luminescent solution (Applygen) according to the manufacturer’s instructions, and protein bands were visualized in a chemiluminescence detector (Tianneng) and quantified by ImageJ software.

### 2.4 qRT-PCR

Total RNA was obtained using Eastep^TM^ Super Total RNA Extraction Kit (Promega), and cDNA was obtained using cDNA Synthesis SuperMix (Novoprotein). Quantitative PCR (qPCR) was performed on ABI Prism^®^ 7,500 Real-Time PCR System (ABI, United States) using SYBR-Green qPCR Master Mix (Low Rox) (MCE) following the manufacturer’s instructions. Briefly, reactions were 10 µL SYBR green Master Mix (×2), 0.2 µM each of forward and reverse primers and 0.5–10 ng/μL cDNA with H_2_O to a total 20 µL volume. The primer sets used are shown in Supplementary Table S2. Reaction conditions were denaturation at 95°C for 5 min, followed by 40 cycles of denaturation at 95°C for 20 s, annealing/extension at 60°C for 40 s. Relative gene expression was calculated based on the threshold cycle (Ct) values and normalization of internal control expression using the 2^−ΔΔCt^ method (Livak and Schmittgen, 2001). The internal control housekeeping gene used in this study was β-actin. Experiments were performed in triplicate and repeated at least three times.

### 2.5 Statistical analysis

Statistical analyses were performed using GraphPad Prism 8.0. The significance of the different treatments was assessed using the Student’s t-test or two-way ANOVA analysis. A *p*-value of < 0.05 was considered statistically significant.

## 3 Results

### 3.1 APG-115 restores p53 expression and activity

**FIGURE 1:**
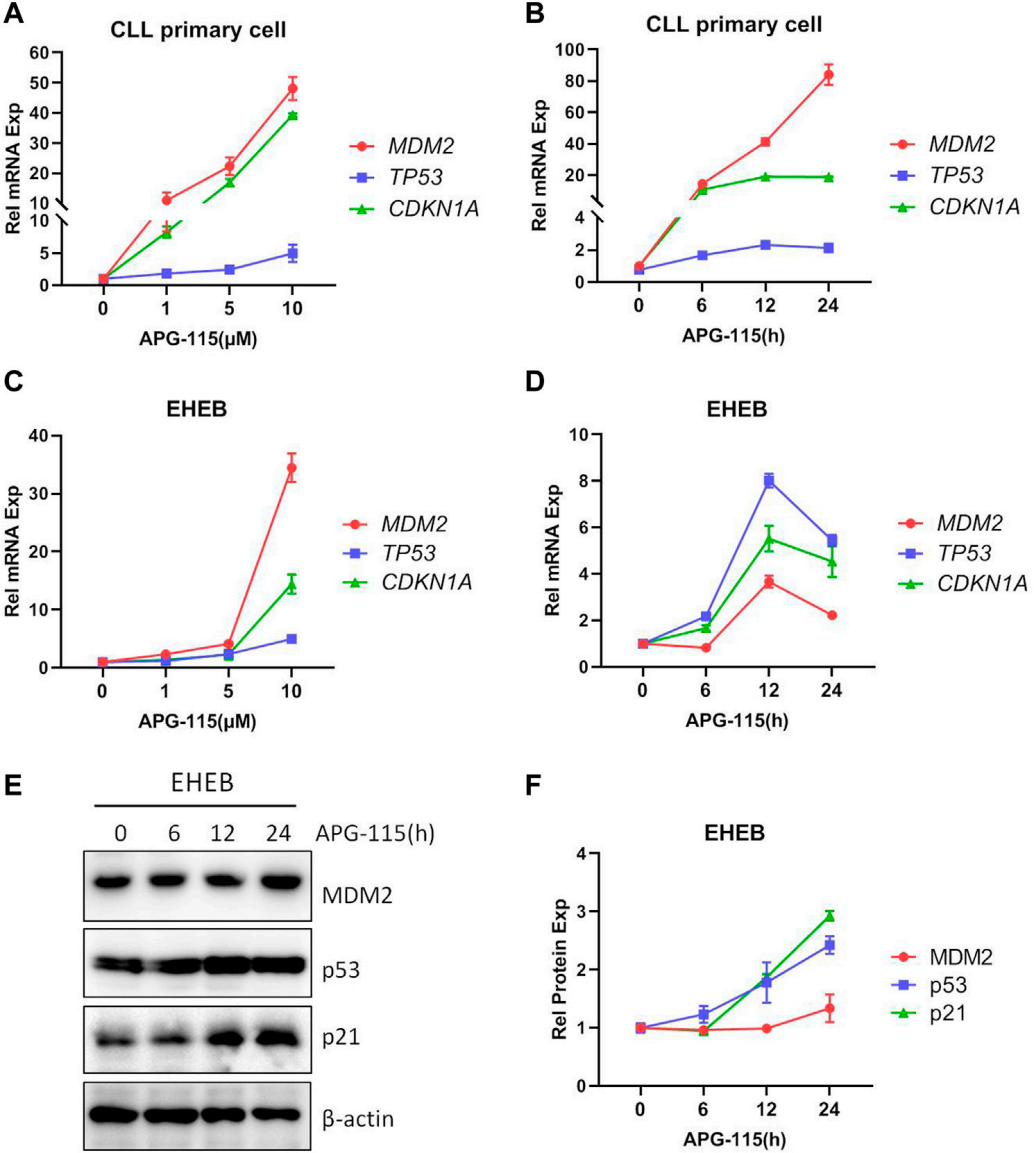
APG-115 restores p53 expression and activity. mRNA expression of *MDM2*, *TP53*, and CDKN1A/p21 were detected in **(A,B)** CLL patient primary cells and **(C,D)** EHEB cells treated with different concentrations APG-115 or different times of APG-115 (10 μM). Relative gene expression was calculated based on the threshold cycle (Ct) values and normalized to β-actin internal control using the 2^−ΔΔCt^ method. Experiments were performed in triplicate and repeated at least three times. The results showed representative primary cells from three CLL patients. **(E)** The protein expressions of MDM2, p53, and p21 in EHEB cells were detected by WB. **(F)** The quantification analysis of protein bands in panel E was performed using ImageJ software; the values of protein bands were divided by the internal control β-actin. The data shown are representative images of three independent experiments.

Given the critical role of p53 in tumorigenesis and cancer development, restoring the function of p53 and inducing p53-dependent apoptosis in tumor cells has been considered an effective strategy for treating cancer. In this study, the effect of novel MDM2 inhibitor APG-115 disrupting the interaction of MDM2-p53 and restoration of p53 expression was first explored in TP53-wild type CLL patient primary cells and EHEB cells. We found that APG-115 treatment upregulated the mRNA expression of MDM2, TP53, as well as its downstream gene cyclin-dependent kinase inhibitor 1 A (CDKN1A), encoding p21 protein (Figures 1A–D). APG-115 treatment also led to protein accumulation of MDM2, p53, and p21 in time- or dose-dependent patterns (Figures 1E, F; Supplementary Figures S1A, B). These results suggested that APG-115 can effectively stabilize p53 and restore its activity.

### 3.2 APG-115 inhibits cell proliferation, induces cell cycle arrest and apoptosis

**FIGURE 2:**
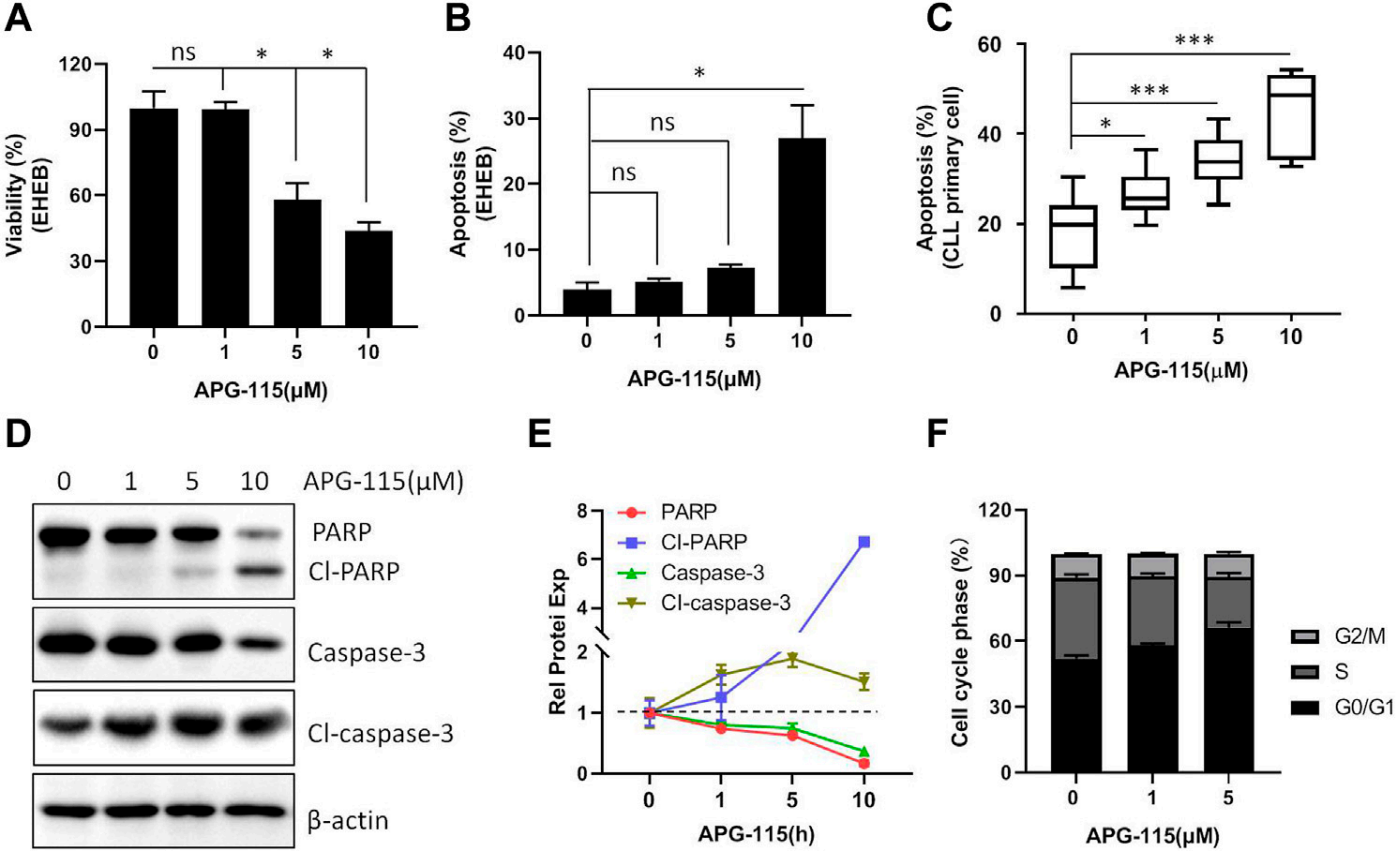
APG-115 inhibits cellular viability and induces apoptosis. **(A)** EHEB cells were treated with different concentrations of APG-115 for 48 h, cell viability was assessed by MTS assay; **(B)** cell apoptosis was detected by flow cytometry. Experiments were performed in triplicate and repeated at least three times. **(C)** Cell apoptosis was detected on CLL patient primary cells (P) (n = 8) cultured with 10 ng CD40L and IL-4 and treated with APG-115 for 48 h. Data was expressed as mean ± standard deviation. Statistical significance was determined using the Student unpaired *t*-test, with Welch’s correction. *, compared to the control group (no APG-115 treatment) *p < 0.05, **p < 0.01, ***p < 0.001. Non-significant results are denoted by ns. **(D)** Protein expression of caspase-3 and PARP was detected by WB in EHEB cells after 12-h treatment with APG-115. β-actin was a control protein. **(E)** Quantification analysis of the protein bands in D using ImageJ software; the values were divided by the internal control β-actin. The data shown are representative images of two independent experiments. **(F)** Statistical histogram of cell cycle in EHEB cells exposed to increasing concentrations of APG-115 for 48 h.

Next, the effect of APG-115 on CLL cell proliferation and apoptosis was studied; we found that the treatment of APG-115 can effectively inhibit cell proliferation in a dose-dependent model (Figure 2A) and is accompanied by increased cell apoptosis in EHEB cells (Figure 2B). Similarly, the pro-apoptotic effect of APG-115 was also observed on CLL patient primary cells, which were cultured in the presence of CD40L and IL-4 to mimic the protective lymph node environment (Figure 2C). Caspase-3 protein plays a critical role in converging upstream apoptosis signals (Boice and Bouchier-Hayes, 2020). An increased level of activated caspase-3 is one of the most common apoptosis markers that have been used to indicate the apoptosis phenotype of cells (Boice and Bouchier-Hayes, 2020). Subsequently, the pro-apoptotic effect of APG-115 was further measured at the molecular level by analyzing the activation of the caspase-3. We found that APG-115 activated caspase-3, which was accompanied by a cleavage of PARP (Figures 2D, E). To determine the mechanism of the anti-proliferative effect of APG-115, cell cycle analysis was performed by flow cytometry. The results showed that exposure of the CLL cells to APG-115 resulted in a significant increase of G0/G1-phase cells and was accompanied by a decrease of S-phase cells, suggesting blocking the transition of the cell cycle from G0/G1 to S phase (Figure 2F). Together, these results demonstrated that APG-115 has a potent anti-proliferation and pro-apoptotic effect in CLL.

### 3.3 APG-115 inhibits anti-apoptotic protein expression and pro-survival signaling pathway activation

**FIGURE 3:**
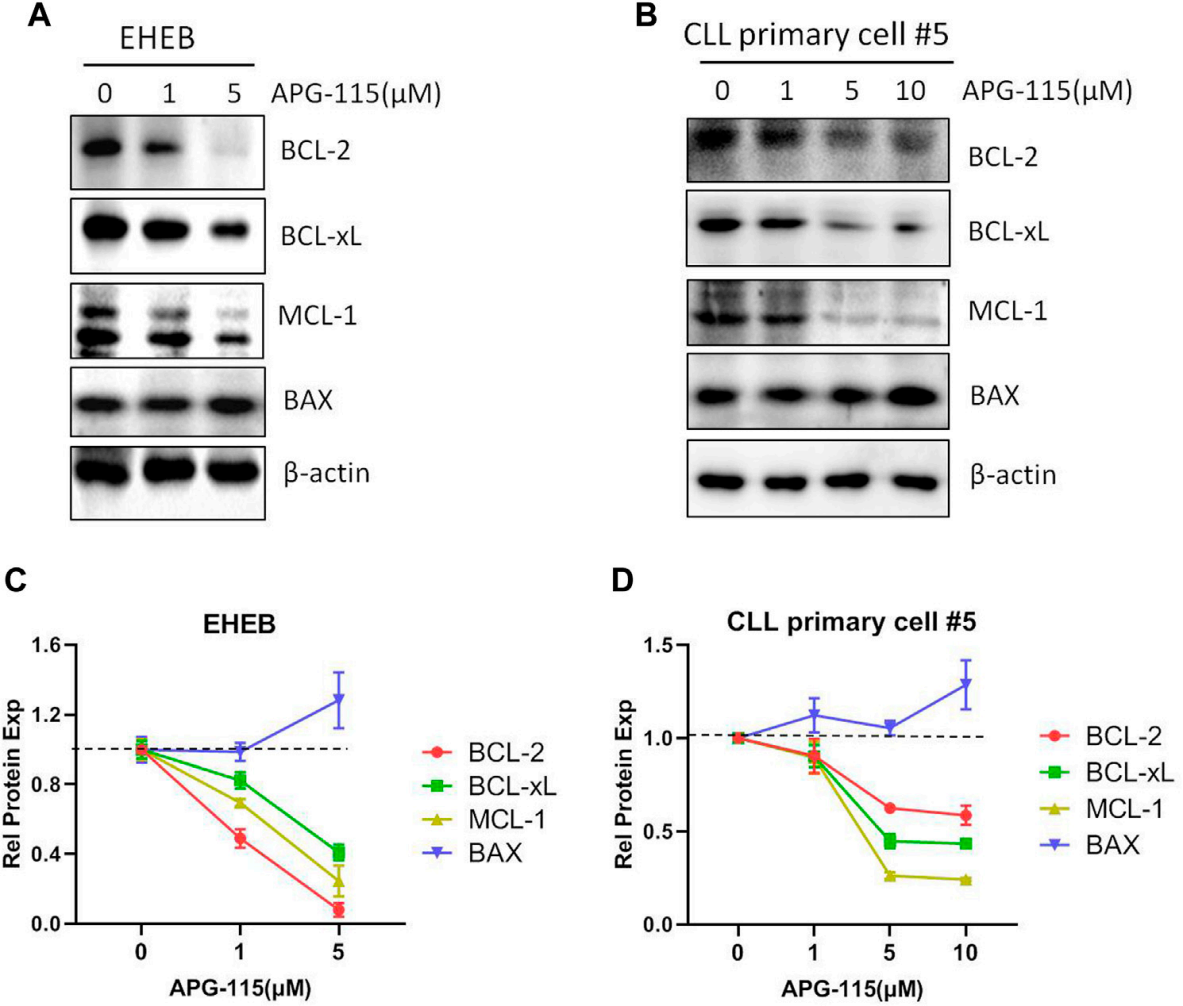
APG-115 inhibits the expression of BCL-2, BCL-xL, and MCL-1. **(A)** EHEB cells and **(B)** CLL patient primary cells were treated with different concentrations of APG-115 for 12 h. The protein expression of BCL-2, BCL-xL, MCL-1, and BAX were detected by WB, and β-actin was the internal control protein. **(C,D)** The quantification analysis of protein bands in **(A,B)** by ImageJ software, a statistical graph showing the value divided by the internal control and compared with the control group without treatment.

**FIGURE 4:**
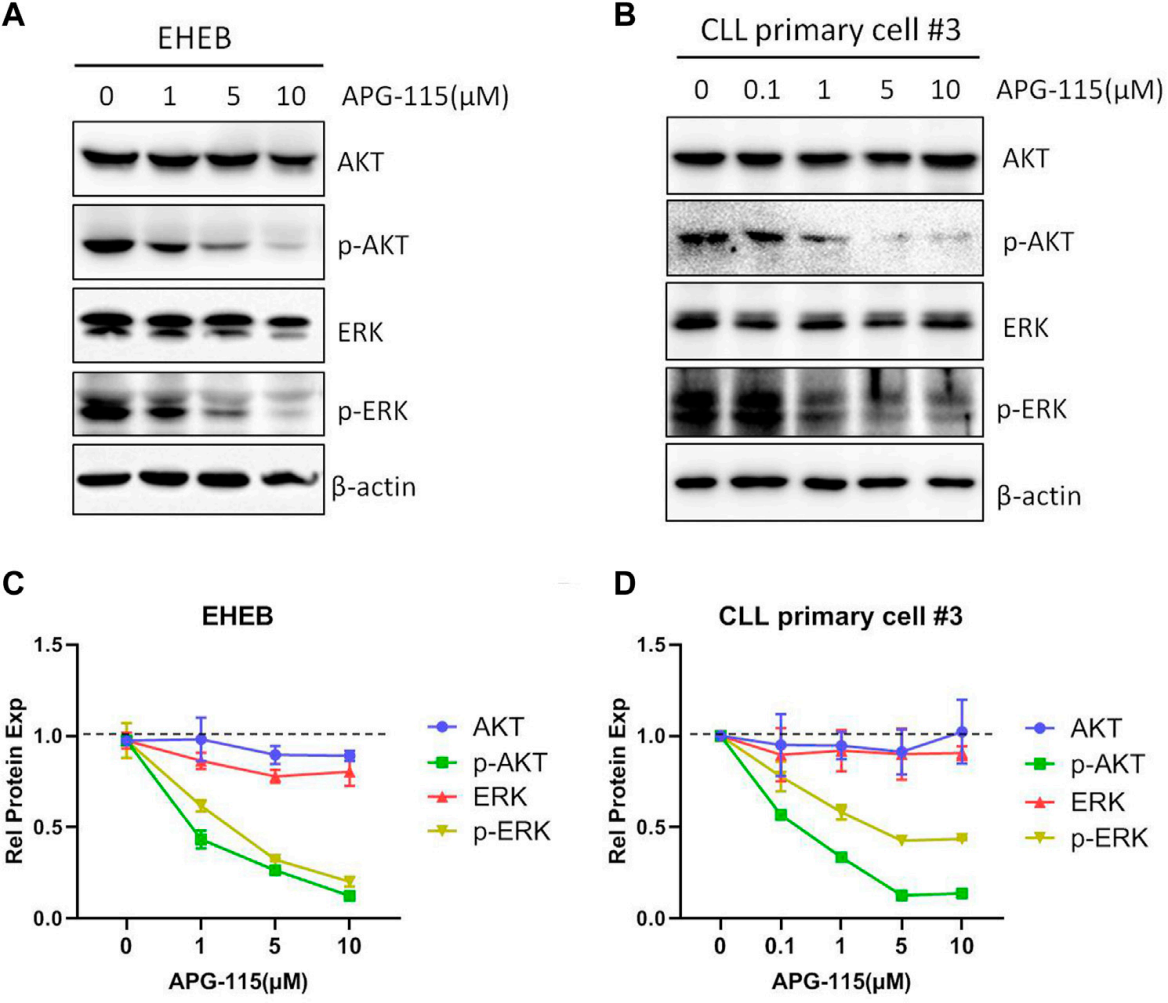
APG-115 suppresses the activation of AKT and ERK signaling pathways **(A)**The protein expression of AKT, p-AKT, ERK, and p-ERK were detected by WB in EHEB cells and **(B)** CLL patient primary cells treated with different concentrations of APG-115 for 12 h. β-actin was the internal reference protein. The results were representatives of primary cells from three CLL patients. **(C,D)** The quantification analysis of protein bands in **(A,B)** by ImageJ software, a statistical graph showing the value divided by the internal reference protein β-actin, and compared with the control group.

BCL-2 family members are essential in initiating and controlling apoptosis at the mitochondrial and ER membrane levels (Kapoor et al., 2020). In CLL cells, the BCL-2 family anti-apoptotic protein is essential for cell survival and anti-apoptosis (Vogler et al., 2009). The regulation of BCL-2 family proteins is the primary mechanism of p53-mediated cell death. p53 transcriptionally activates BCL-2 family pro-apoptotic members and inhibits the expression of anti-apoptotic BCL-2, BCL-xL and MCL-1, in addition, p53 also promotes mitochondria-mediated apoptosis by directly associating with multiple BCL-2 family proteins (Pan et al., 2017; Hao et al., 2023). Thus, we evaluated the effect of APG-115 on the expression of pro-apoptotic proteins in CLL cells. We found that APG-115 significantly inhibited the expression of anti-apoptotic proteins MCL-1, BCL-xL, and BCL-2 in a dose-dependent model in both the EHEB cell line and CLL patient primary cells (Figure 3). In contrast, APG-115 had no inhibitory effect on pro-apoptotic protein BAX expression but slightly promoted its expression (Figure 3). AKT and ERK are the central functional target kinases in PI3K/AKT and RAF/MEK/ERK signaling pathways, whose activation is closely related to CLL cell survival and anti-apoptosis (Kawauchi et al., 2002; Ringshausen et al., 2002; Chen et al., 2016; Chen et al., 2019). We thus evaluated the effect of APG-115 on these pro-survival signals in CLL cells. We found that APG-115 significantly inhibited the activation of AKT and ERK in both EHEB cells and CLL patient primary cells in dose-dependent manners (Figure 4). Taken together, these data provided a mechanism for APG-115 anti-proliferation and pro-apoptotic efficiency in CLL.

### 3.4 APG-115 synergizes ABT-199 to induce CLL cell apoptosis

**FIGURE 5:**
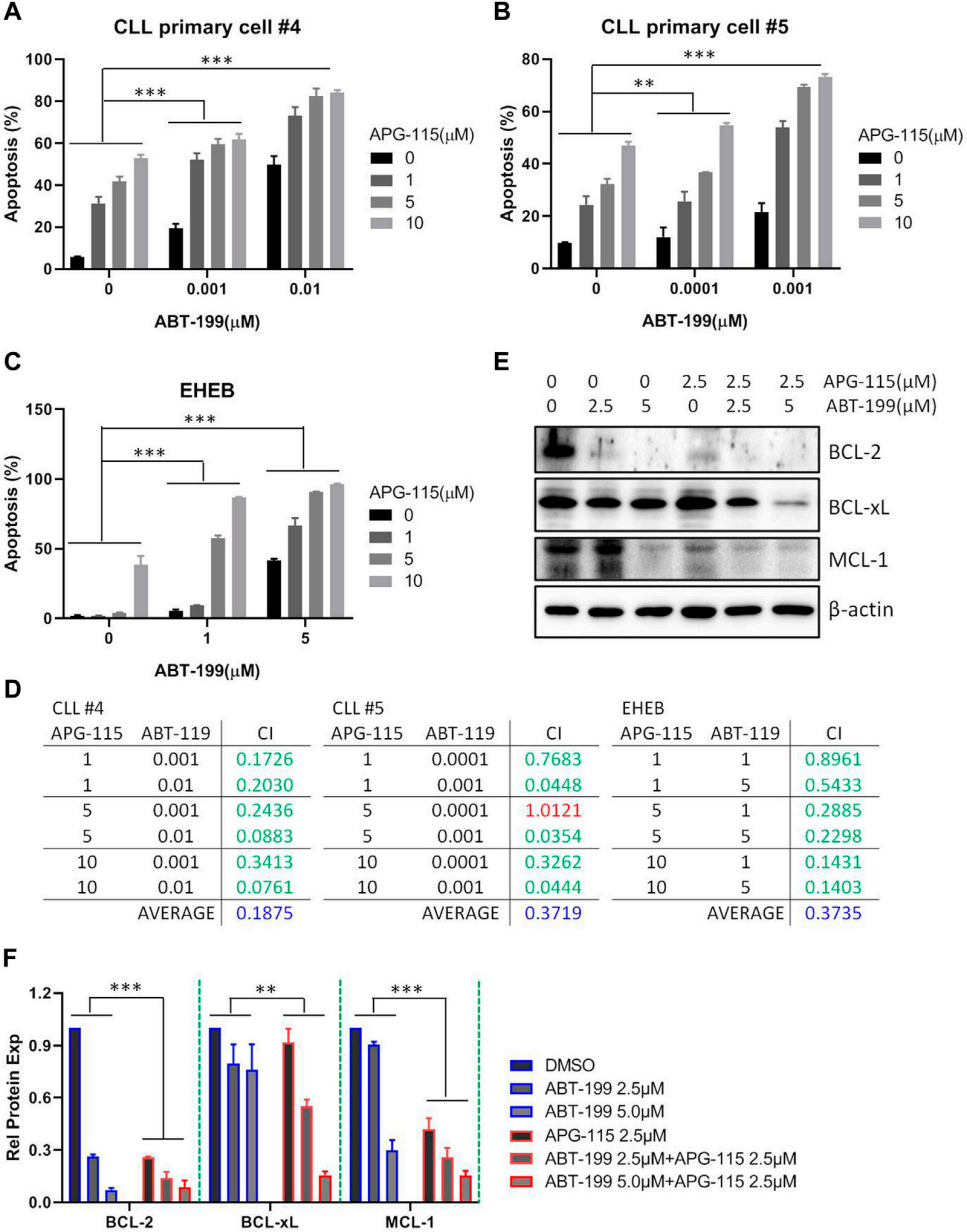
APG-115 synergizes ABT-199 to induce cell apoptosis in CLL **(A,B)** Cell apoptosis was detected by flow cytometry in CLL patient primary cells, and **(C)** EHEB cells that were treated with described concentrations of APG-115 and ABT-199 for 48 h. The figure shows the representatives of three independent experiments, and the results are expressed as mean ± standard deviation. **(D)** The synergic effect was evaluated using the Compusyn software. The criteria for the Combination index (CI) value are as follows: <0.1, Very strong synergism; 0.1–0.3, Strong synergism; 0.3–0.7, Synergism; 0.7–0.85, Moderate synergism; 0.85–0.9, Slight synergism; 0.90–1.1, Nearly additive; >1.1 antagonism. In green, shows synergistic combinations. In red, shows additive combinations. In blue, shows average synergy in all combinations. All concentrations are in μM. **(E)** The effect of combination of APG-115 and ABT-119 on BCL-2, BCL-xL, and MCL-1 expression in EHEB cells. The data shown are representative images of three independent experiments. **(F)** The quantification analysis of protein bands in **(E)** by ImageJ software, the graph shows the value divided by the internal reference β-actin and compared with the control group without treatment. Statistical significance was determined using two-way ANOVA analysis with the Geisser-Greenhouse correction. A *p*-value of < 0.05 was considered statistically significant.*p < 0.05, **p < 0.01, ***p < 0.001.

Given the potent pro-apoptotic effect of MDM2 inhibitors in inducing cell death, various combination strategies with MDM2 inhibitors are currently being investigated in the preclinical or clinic (Konopleva et al., 2020). Here, we assessed the combination of APG-115 with BCL-2 inhibitor ABT-199 (also known as venetoclax), one of the most active chemotherapeutic agents in the therapy of CLL (Roberts et al., 2016). We found that the combination of both agents could significantly induce cell apoptosis in the EHEB cell line and CLL patient primary cells protected by the mimic environment in a dose-dependent manner (Figures 5A–C). This combination has a synergistic pro-apoptotic effect based on the analysis of the combination index (CI), a marker to reflect the drug interaction, which was evaluated by using the Compusyn software (Figure 5D). To investigate the mechanism underlying the enhanced pro-apoptotic activity of this combination, we next assessed the effect on BCL-2 family anti-apoptotic protein expression. Western blot analysis revealed that the combination of APG-115 and ABT-119 enhanced the inhibition of BCL-2 expression and suppressed the expression of MCL-1 and BCL-xL (Figures 5E, F). Particularly for BCL-xL, the treatment of ABT-199 alone has no significant effect on BCL-xL expression compared to the control. In contrast, the combination exerted a significant inhibiting effect on BCL-xL expression. These data verified that APG-115 can combine with ABT-199 and synergistically induce cell apoptosis in CLL. These results afforded a sound scientific rationale to further evaluate the combination of APG-115 with ABT-199 in clinical therapy.

## 4 Discussion

Although recent therapeutic strategies have significantly improved the survival of CLL patients, CLL remains incurable. The effort to explore effective treatment approaches remains necessary. The present study reports that a novel MDM2 inhibitor, APG-115, has a potent anti-tumor effect in CLL. APG-115 treatment restored p53 stabilization and its activity, which thus effectively inhibits cell proliferation, arrests cell cycle and induces cell apoptosis, and the combination with ABT-199 demonstrates a synergistic pro-apoptotic effect, suggesting a potential application of APG-115 in CLL therapy.

Tumor suppressor protein p53 plays a pivotal role in triggering cell apoptosis and thus inhibits the occurrence of tumors. However, in cancer cells, the p53 level is typically deficient (Shangary and Wang, 2008). MDM2 not only controls the transcription and expression of p53 but also regulates p53 protein degradation by its ubiquitination ligase activity (Scheffner et al., 1990; Tommasino et al., 2003). Thus, using MDM2 inhibitor to restore the native tumor-suppressing functions of p53 and activate p53-dependent apoptosis in malignant cells has been considered a desirable treatment for cancer therapy (Saha et al., 2010). Currently, MDM2 inhibitors have been developed and evaluated in several malignancies. The preclinical and clinical studies have supported the viability of the MDM2 inhibitors-related approach to mediate anti-tumor activity (Andreeff et al., 2016; Tisato et al., 2017; Konopleva et al., 2020). In CLL cells, MDM2 has been reported to be over-expressed in 28% of CLL cases, suggesting an excellent potential for inducing cell death by inhibiting MDM2 activity. Consistently, MDM2 inhibitor Nutlin-3a was reportedly sensitive to CLL patients (Saddler et al., 2008). In this study, we verified that a novel MDM2 inhibitor, APG-115, can interrupt the association between p53 and MDM2, thus leading to the accumulation of p53 (Figure 1E). The restored p53 activity regulates the transcription and expression of many targeted genes such as CDKN1A, P27KIP1, and MDM2, which functionally regulate cell cycle and proliferation (Figures 1A–D). The effect of APG-115 on cell apoptosis, proliferation, and cell cycle arrest was evaluated in this study. We found that APG-115 can effectively inhibit cell viability and induce cell apoptosis in CLL (Figures 2A–C), supported by the progressive cleavage of PARP and activation of caspase-3 (Figures 2D, E), which is consistent with the observation in DLBCL, AML, and ALL (Luo et al., 2020; Zhai et al., 2023). Besides, APG-115 also effectively arrested the cell cycle at G0/G1 stage (Figure 2F). Mechanistically, APG-115 affected the expression of several cell cycle regulation genes including CDKN1A, GADD45A, and P27KIP1 (Figures 1A–D; Supplementary Figure S2). The expression of these genes provided the mechanistic explanation for APG-115 arresting the cell cycle. Taken together, APG-115 inhibited cell proliferation and induced cell cycle arrest, eventually leading to CLL cell death, suggesting a potent efficiency and potential application of APG-115 in treating hematologic malignancies.

Previous studies showed that the status of p53 is a major determinant of therapeutic responsiveness to MDM2 inhibitors in various cancers/leukemia (Kojima et al., 2006; Saddler et al., 2008; Luo et al., 2020; Zhai et al., 2023). APG-115 was recently demonstrated to be sensitive to cancer cells with wild-type p53, whereas it is less effective in p53 mutant DLBCL, AML, and ALL cells (Luo et al., 2020; Zhai et al., 2023). Our data showed that APG-115 can effectively induce apoptosis for the CLL cells with wild-type p53 (Figure 2). However, it is notable that APG-115 also has a moderate effect on cell viability and apoptosis in p53-mut CLL cells such as MEC-1 cells (Supplementary Figures S3A, B). This probably is because these cells have functional p53, although their TP53 gene is mutated. We consistently detect p53 expression at mRNA and protein levels in MEC-1 cell, and found that the p53 protein molecular size is slightly smaller than the wild-type p53 (Supplementary Figures S3C, D). A similar result was also observed previously (Pozzo et al., 2013). In addition, this study also verified that APG-115 inhibits the expression of BCL-2, BCL-xL, and MCL-1 (Figure 3), consistent with the negative regulatory function of p53 on the expression of a myriad of BCL-2 family anti-apoptotic proteins (Pan et al., 2017; Hao et al., 2023). In addition to the inhibition on BCL-2 family anti-apoptotic proteins expression, we noticed that APG-115 has an inhibitory effect on AKT and ERK pro-survival signaling pathways activation in CLL cells (Figure 4). This is probably because the activation of p53 can negatively regulate the AKT and ERK signaling pathways (Pan et al., 2017). Collectively, these results suggested that CLL patients can benefit from the treatment with APG-115.

MDM2 inhibitor has been considered a potential drug in cancer therapeutics due to the activation of p53 to enhance cancer cell sensitivity to apoptosis. More and more studies have demonstrated that MDM2 inhibitors can combine with various agents, including PI3K/AKT, RAF/MEK/ERK signaling pathways inhibitors and CDK inhibitors, anti-CD20 antibodies, and anti-PD-L1/PD-1 agents to induce apoptosis in various types of cancers/leukemia cells (Kojima et al., 2007; Kojima et al., 2008; Herting et al., 2016; Laroche et al., 2017; Konopleva et al., 2020; Roulleaux Dugage et al., 2021). The combination of MDM2 and BCL2 inhibitors is also being explored in several types of cancer cells (Hoffman-Luca et al., 2015; Lehmann et al., 2016; Konopleva et al., 2020). Most recently, APG-115 combined with BCL-2 inhibitor APG-2575 exhibited synergistic anti-proliferative and pro-apoptotic activities in DLBCL, AML, and ALL cells (Luo et al., 2020; Zhai et al., 2023). In this study, we demonstrated that APG-115 significantly enhanced the efficiency of ABT-199 to promote CLL cell death and elicited synergistic anti-leukemic activity (Figure 5). The synergistic effect was also observed in CLL patient primary cells protected by a mimic environment (Figures 5A, B). This combination enhanced the inhibitory effect on the expression of BCL-2 family anti-apoptotic proteins, suggesting a determinant role in inducing cell death. p53 and BCL-2 represent two important regulators in cell apoptosis. The combining efficiency suggests a potential of simultaneously targeting p53 and BCL-2 and provides a rational basis for clinical testing of this therapeutic approach in CLL.

**FIGURE 6:**
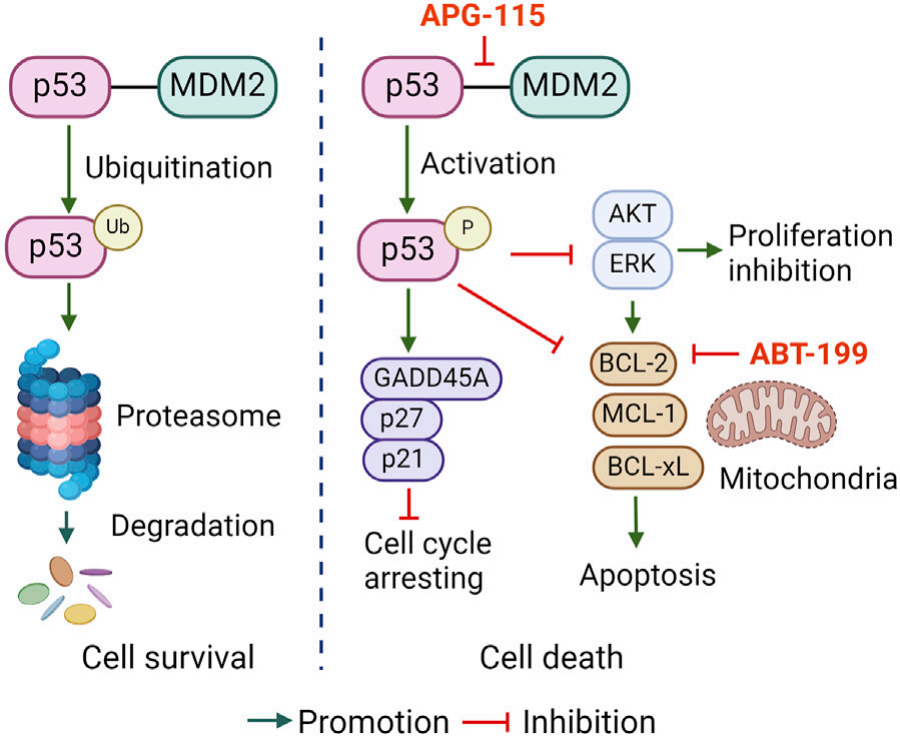
The proposed pro-apoptotic mechanism of APG-115 in CLL.

Taken together, this study reports that APG-115 effectively restored p53 activation thus induced cell cycle arrest, inhibited multiple pro-survival signals activation, and BCL-2 family anti-apoptotic protein expression, which collectively affords a mechanistic explanation for APG-115 effectiveness in inducing CLL cell apoptosis (Figure 6). The synergistic effect of APG-115 with ABT-199 suggested a potential combination application in CLL therapy.

## Data Availability

The original contributions presented in the study are included in the article/Supplementary Materials, further inquiries can be directed to the corresponding author.
