# Supplementary material for: MDM2 inhibitor APG-115 synergizes with ABT-199 to induce cell apoptosis in chronic lymphocytic leukemia
Source: Front Pharmacol. 2024 Jul 31;15:1441383. doi: 10.3389/fphar.2024.1441383 (PMC11321975; doi:10.3389/fphar.2024.1441383)
Supplement: Supplementary file 1 [file DataSheet1.docx]

**Supplementary data**

**Materials and methods**

**Supplementary Table 1** (Table S1) Clinical data available for CLL patients

| **CLL Patients** | **Sex** | **Age** | **Cytogenetics** |
| --- | --- | --- | --- |
| P1 | M | 79 | TP53, chromosome 12 and 13 normal |
| P2 | M | 70 | ATM, NOTCH1, SF3B1 genes normal, p53 deletion (35%) |
| P3 | M | 71 | RB1(13q14) gene, ATM (11q22.3) gene normal |
| P4 | M | 70 | RB1(13q14) gene deletion (87%), ATM (11q22.3) gene deletion, No t(11:14) translocation formed IGH/CCND1 fusion gene, Notch1 (p.P2138Rfs*110) mutation. TP53 (17p13.1) gene no deletion |
| P5 | M | 92 | NOTCH1 gene p.P2138Rfs*110 frameshift mutation (33.0%), SF3B1 gene p.N626S missense mutation (4.2%), ATM gene deletion; BIRC3, BTK, MYD88, PLCG2, TP53 genes normal |
| P6 | M | 82 | RB1(13q14) deletion, D13S25(13q14) deletion, ATM(11q22) deletion |
| P7 | M | 43 | No mutation in ATM, BTK, BIRC3, MYD88, NOTCH1, PLCG2, SF3B1 and TP53 genes, IGH/ccnd1 normal, no 17q deletion |
| P8 | F | 73 | homozygous deletion in 13q14 |

**Supplementary Table 2** (Table S2) qPCR primers

| **Gene** | **Forward 5'->3'** | **Reverse 5'->3'** |
| --- | --- | --- |
| MDM2 | GAATCATCGGACTCAGGTACATC | TCTGTCTCACTAATTGCTCTCCT |
| TP53 | CAGCACATGACGGAGGTTGT | TCATCCAAATACTCCACACGC |
| CDKN1A (p21) | TGTCCGTCAGAACCCATGC | AAAGTCGAAGTTCCATCGCTC |
| P27KIP1 | ATCACAAACCCCTAGAGGGCA | GGGTCTGTAGTAGAACTCGGG |
| GADD45A | GAGAGCAGAAGACCGAAAGGA | CAGTGATCGTGCGCTGACT |
| β-actin | AAAGACCTGTACGCCAACAC | GTCATACTCCTGCTTGCTGAT |

**Results**

**Supplementary Figure 1**


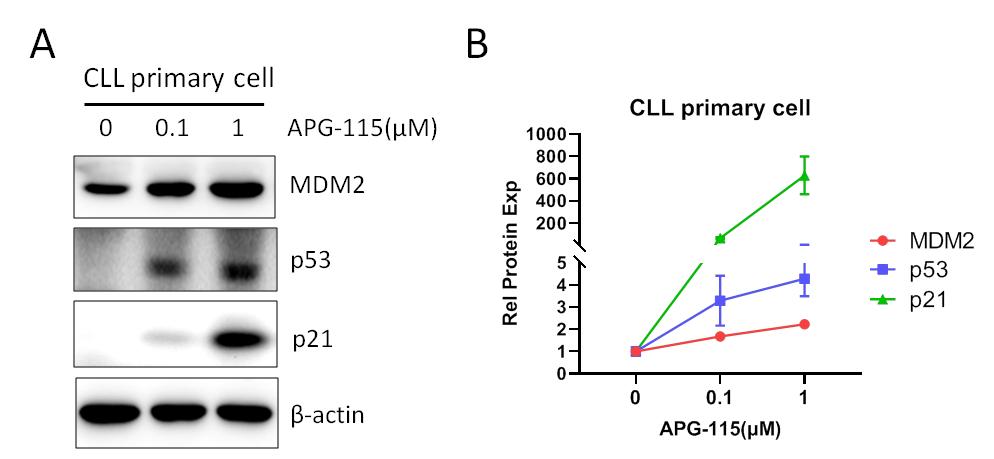


**Figure 1** **APG-115 restores p53 expression and** **activity** (A) CLL patient primary cells were treated with different concentrations of APG-115 for 24 hours, the protein expressions of MDM2, p53, and p21 were detected by WB, β-actin was the internal reference protein. (B) The quantification analysis of protein bands in panel A was performed using ImageJ software; a statistical graph showing the value divided by β-actin, and compared with the control group.

**Supplementary Figure 2**


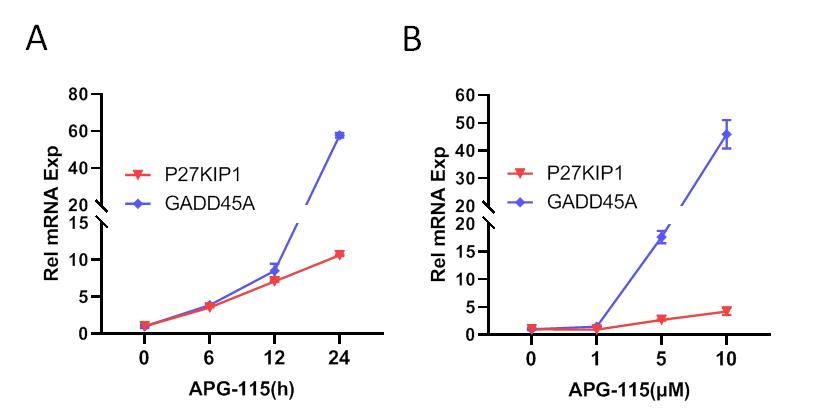


**Figure 2 APG-115 increases p53-dependent gene expression.** (A) CLL cells treated with different times or concentrations of APG-115. The relative mRNA expression of P27KIP1 and GADD45A genes was detected by qPCR. Experiments were performed in triplicate and repeated at least three times. The results were expressed as the mean ± SD.

**Supplementary Figure 3**


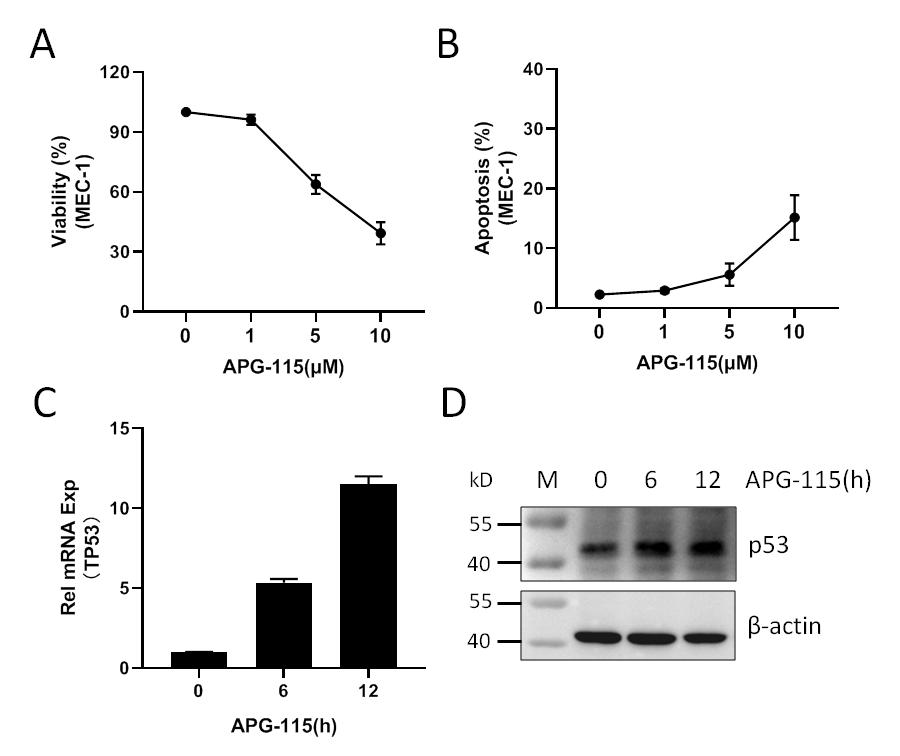


**Figure 3 APG-115 inhibits MEC-1 cell viability and induces apoptosis.** (A) MEC-1 cells were treated with different concentrations of APG-115 for 48 hours, cell viability was assessed by the MTS Assay; (B) cell apoptosis was detected by flow cytometry. Experiments were performed in triplicate and repeated at least three times. (C) The mRNA expression of *TP53* gene was detected in MEC-1 cell treated with APG-115 (10μM). Relative gene expression was calculated as Figure 1. (D) The protein expressions of p53 in MEC-1 cells were detected by WB. β-actin was internal control.
